# Development and validation of a prediction model based on a nomogram for tuberculous pleural effusion

**DOI:** 10.3389/fmed.2025.1589406

**Published:** 2025-07-18

**Authors:** Suli Liu, Yao Yang, Dongmei Wang, Lijuan Gao, Jiangyue Qin, Yanqiu Wu, Diandian Li, Xiaohua Li, Mei Chen, Hao Wang, Yongchun Shen, Fuqiang Wen, Fangying Chen

**Affiliations:** ^1^Division of Pulmonary Diseases, State Key Laboratory of Biotherapy of China, Department of Respiratory and Critical Care Medicine, West China Hospital, Sichuan University, Chengdu, China; ^2^State Key Laboratory of Respiratory Health and Multimorbidity, West China Hospital, Sichuan University, Chengdu, China; ^3^Department of Medical Ultrasound, West China Hospital, Sichuan University, Chengdu, China; ^4^Department of Respiratory and Critical Care Medicine, Sixth People’s Hospital of Chengdu, Chengdu, China; ^5^Key Laboratory of Acupuncture for Senile Disease (Chengdu University of TCM), School of Medical and Life Sciences, Chengdu University of Traditional Chinese Medicine, Ministry of Education, Chengdu, China; ^6^Department of Tuberculosis, The Third People’s Hospital of Tibet Autonomous Region, Lhasa, China

**Keywords:** tuberculosis, tuberculous pleural effusion, clinical prediction model, diagnosis, nomogram

## Abstract

**Background:**

Diagnosing tuberculous pleural effusion (TPE) is challenging. There is a lack of cross-sectional lateral comparisons among TPE prediction models.

**Objectives:**

We aimed to develop and validate a novel TPE prediction model and compare its diagnostic performance with that of existing models.

**Methods:**

Patients with pleural effusion were included in the training, testing, and external validation sets. Variable selection strategies included LASSO and logistic regression. The discriminability, calibration, and clinical efficacy of the prediction model were estimated in the three sets. The performance of the model was compared with that of two existing prediction models.

**Results:**

Fever, tuberculosis interferon-gamma release assays, pleural adenosine deaminase, the pleural mononuclear cell ratio, the ratio of pleural lactate dehydrogenase to pleural adenosine deaminase, pleural carcinoembryonic antigen, and pleural cytokeratin 19 fragment were selected to establish the prediction model. The AUCs were 0.931 (0.903–0.958), 0.856 (0.753–0.959), and 0.925 (0.867–0.984) in the training, testing, and external validation sets, respectively. The AUCs of the two existing prediction models were 0.793 (0.737–0.850) and 0.854 (0.816–0.892). The calibration curves revealed that this model had good consistency. Decision curve analysis revealed the acceptable clinical benefit of this model.

**Conclusion:**

Compared with the existing models, the TPE prediction model developed in this study demonstrated good diagnostic performance.

## 1 Introduction

Tuberculosis remains the leading cause of death among infectious diseases globally, ranking 13th among the main causes of death globally (1). According to the World Health Organization, approximately 25% of the world’s population is infected with *Mycobacterium tuberculosis*. The global burden of disease (GBD) in 2021 estimated 9.4 million new tuberculosis cases and 1.35 million deaths (2). Pleural tuberculosis is the most common type of extrapulmonary tuberculosis and the main cause of pleural effusion. The European Respiratory Society reported that 6% of pleural effusion among adults is attributed to tuberculous pleural effusion (TPE) (3). However, this proportion varies significantly across different tuberculosis-endemic regions. Available documentation indicates that in South Africa, India, China, and Nigeria, the proportions of TPE among all pleural effusion cases are 82.4, 23.5, 40, and 32.9%, respectively. In contrast, this proportion ranges from 0% to 5.9% in non-endemic areas (4). A nationwide survey on the causes of pleural effusion in hospitalized patients in China revealed that 12.3% of pleural effusion was attributable to tuberculosis (5). The China tuberculosis surveillance system indicates that the incidence rate of tuberculosis is higher in rural, central, and western regions than in urban and eastern regions, which also reflects regional differences in TPE epidemiology (6, 7). TPE may result in complications such as empyema, pleural thickening, pleural calcification, chylothorax, or bronchopleural fistula, all of which can impair respiratory function and quality of life. In addition, a US study followed 141 military personnel with a positive tuberculin skin test and pleural effusion from 1940 to 1944. Although the pleural effusion resolved spontaneously within 2–4 months without treatment, 65% of the patients developed active tuberculosis within 2 years (8, 9). Therefore, it is crucial to identify TPE early.

Early detection of TPE may reduce the risk of the above complications. The diagnosis of TPE relies on acid-fast staining, *Mycobacterium tuberculosis* culture, or pleural biopsy through thoracoscopy, which have limitations such as extended duration, a low positive rate, and invasiveness. Studies have reported that the diagnostic sensitivity of thoracoscopy can reach 90%–100%, whereas the sensitivity of pleural effusion acid-fast staining and mycobacterial culture ranges from 10% to 20% and 20% to 45%, respectively (10–13). However, the clinical applicability of thoracoscopy is relatively low because of its cost and invasiveness. Some biochemical indicators, such as tuberculosis interferon-gamma release assays (TB-IGRA), pleural effusion adenosine deaminase (pADA), pleural effusion lactate dehydrogenase (pLDH), the pLDH/pADA ratio, and pleural effusion cytological categorization, may serve as auxiliary diagnostic markers. However, the sensitivity and specificity of utilizing a single marker to differentiate TPE patients from non-TPE patients require improvement. Furthermore, different types of PEs may exhibit similar pathophysiologies and laboratory indicator changes, so multiple indicators are needed to classify PEs from various perspectives to improve diagnostic accuracy.

Clinical prediction models are statistical models that use multiple variables to predict diseases, prognoses, or other medical conditions (14). There are several TPE prediction models based on single clinical and combined biomarkers (15–17). However, owing to differences in research populations and modeling approaches, these models exhibit significant heterogeneity. Additionally, lateral comparisons among these models are lacking. Therefore, this study intends to develop a combined diagnostic model based on laboratory and clinical features and validate current prediction models on the basis of our data to compare their prediction performance.

## 2 Materials and methods

### 2.1 Derivation population and study design

We retrospectively collected data from patients with pleural effusion at West China Hospital from January 2020 to December 2022. The inclusion criteria were as follows: (1) ≥ 18 years of age and (2) pleural effusion diagnosed by either ultrasonography or chest CT. The exclusion criteria were as follows: (1) unknown etiology of PE, (2) transudative pleural effusion according to Light’s criteria, and (3) incomplete clinical data. The diagnostic criteria for TPE were as follows: (1) a pleural effusion or pleural biopsy sample that was positive for acid-fast staining/culture of *Mycobacterium tuberculosis* or (2) the discovery of a caseous necrotizing granuloma in the pleural biopsy sample (10). Patients were classified into TPE and non-TPE groups. Non-TPE included pleural effusion caused by infection, cancer, or other reasons according to well-accepted criteria (18). The derivation population was randomly divided into a training set and a testing set at a ratio of 8:2.

This report is compliant with the Strengthening the Reporting of Observational Studies in Epidemiology (STROBE) statement. The STROBE checklist is provided in Supplementary material 1. This study was approved by the institutional Ethics Committee of West China Hospital (WCH 2024–1108) and was conducted in accordance with the Helsinki Declaration. Written informed consent was exempted by the ethics committee because this study was retrospective without any risks to the patients.

### 2.2 External validation population

We retrospectively collected data from patients with pleural effusion at West China Hospital from January 2023 to October 2024 to validate the model performance externally. The same laboratory examination testing protocol was used for both the training and the external validation cohorts. The inclusion criteria, exclusion criteria, and diagnostic criteria of the external validation set were the same as those of the derivation population.

### 2.3 Data collection

The laboratory examination method was consistent and standardized across all patients and timepoints. The following clinical data were extracted from the hospital information system of West China Hospital: (1) demographic characteristics: age, sex, and smoking history, (2) disease characteristics: clinical symptoms (fever, cough with sputum, chest pain, hemoptysis, and dyspnea), and (3) laboratory examination: TB-IGRA, serum hemoglobin concentration (HGB), platelet count (PLT), white blood cell count (WBC), neutrophil count, lymphocyte count, neutrophil/lymphocyte ratio (NLR), procalcitonin (PCT), C-reactive protein (CRP), interleukin-6 (IL-6), B-type natriuretic peptide (BNP), erythrocyte sedimentation rate (ESR), fibrinogen degradation products (FDP), D-dimer, pleural effusion mononuclear and multinuclear cell ratio, logarithm of the ratio of pleural effusion mononuclear cells to multinuclear cells (lnRMMPE), the concentration of pADA, serum and pleural effusion total protein (s/p TP), lactate dehydrogenase (s/p LDH), carcinoembryonic antigen (s/p CEA), carbohydrate antigen 125 (s/p CA125), carbohydrate antigen 19–9 (s/p CA199), carbohydrate antigen 153 (s/p CA153), cytokeratin 19 fragment (s/p CYFRA21-1), and neuron-specific enolase (s/p NSE). The ratio of indicators in the pleural effusion fluid to those in the serum and the ratio of pLDH to pADA were also calculated (19, 20). The continuous variable pADA concentration was transformed into a binary variable on the basis of the generally accepted cutoff value of 40 U/L (21). Multiple imputation by chained equations (MICE) was used to handle variables with less than 20% missing data. Four variables with more than 20% missing data were excluded: BNP (missing rate of 30.9%), ESR (missing rate of 76.2%), sCA153 (missing rate of 59.3%), and pCA153 (missing rate of 62.4%).

### 2.4 Statistical analysis

Continuous variables were tested for normality via the Shapiro–Wilk test. Normally distributed continuous variables are expressed as the mean ± standard deviation, and the difference between groups was estimated by an independent sample *t*-test. Abnormally distributed continuous variables are expressed as medians with first and third quartiles, while the Mann–Whitney U test was used for comparisons between groups. Categorical variables are expressed as frequencies, and the chi-square test was used for comparisons between groups.

In the training set, univariate logistic regression analysis was used to screen the potential variables according to a two-sided *P* < 0.05. Least absolute shrinkage and selection operator (LASSO) regression analysis was used to further select prediction parameters without multicollinearity and to prevent overfitting. The continuous variables were converted into binary variables to facilitate clinical application and statistical optimization according to the cutoff value corresponding to the maximally selected Wilcoxon rank statistics method (MSRS). The selected variables were incorporated into the nomogram. Receiver operating characteristic (ROC) curves, precision–recall (PR) curves, calibration curves, decision curve analysis (DCA), and clinical impact curve (CIC) analyses were performed to determine the discrimination, consistency, and practicability of the models. The area under the precision–recall curve (PR AUC) was used to assist in evaluating the discrimination of the model in the case of class imbalance. The Hosmer–Lemeshow (HL) test was used to assess the goodness-of-fit of the model, with a *p*-value greater than 0.05 indicating goodness of fit. Two existing TPE models were applied to our dataset to compare the model performance (16, 17). All the statistical analyses were performed via R 4.3.2. The R packages used included moonBook, autoReg, mice, caret, glmnet, foreign, rms, pROC, Hmisc, rmda, nomogramFormula, nomogramEx, PRROC, and shiny.

## 3 Results

### 3.1 Clinical characteristics of the derivation population

In total, 537 patients (101 TPE patients and 436 non-TPE patients) were included in the derivation dataset. Among the non-TPE patients, 233 had MPE, 181 had PPE, and 22 had exudate pleural effusion caused by other reasons. The inclusion flowchart is shown in Supplementary material 2. The derivation dataset was randomly divided into a training set (*N* = 432) and a testing set (*N* = 105) at a ratio of 8:2. No significant difference was observed between the two sets of variables (Supplementary material 3). The baseline characteristics of the derivation population are shown in Table 1, and the details of all the variables are shown in Supplementary material 4.

**TABLE 1:** Baseline characteristics of derivation population.

Variables	Training set			Testing set		
	non-TPE (*n* = 346)	TPE (*n* = 86)	*p*	non-TPE (*n* = 90)	TPE (*n* = 15)	*p*
Sex			0.176			0.872
Female	136 (39.31%)	27 (31.40%)		38 (42.22%)	6 (40.00%)	
Male	210 (60.69%)	59 (68.60%)		52 (57.78%)	9 (60.00%)	
TB-IGRA			0.000			0.000
Negative	316 (91.33%)	25 (29.07%)		82 (91.11%)	8 (53.33%)	
Positive	30 (8.67%)	61 (70.93%)		8 (8.89%)	7 (46.67%)	
Fever			0.000			0.128
No	302 (87.28%)	54 (62.79%)		75 (83.33%)	10 (66.67%)	
Yes	44 (12.72%)	32 (37.21%)		15 (16.67%)	5 (33.33%)	
pADA ≥ 40 (U/L)			0.000			0.000
No	311 (89.88%)	63 (73.26%)		87 (96.67%)	10 (66.67%)	
Yes	35 (10.12%)	23 (26.74%)		3 (3.33%)	5 (33.33%)	
Age (years)	64.00 (53.00–72.00)	57.50 (46.00–74.00)	0.153	64.00 (54.00–69.00)	56.00 (46.50–65.00)	0.140
pADA (IU/L)	8.95 (6.60–14.10)	24.75 (10.10–42.90)	0.000	10.10 (7.70–15.20)	25.70 (9.10–46.70)	0.030
pLDH (IU/L)	320.00 (182.00–639.00)	229.00 (146.00–400.00)	0.014	287.00 (200.00–557.00)	234.00 (169.50–314.00)	0.171
pLDH/pADA	36.33 (22.82–58.33)	12.41 (7.53–21.73)	0.000	32.03 (20.00–64.42)	11.00 (8.55–22.37)	0.000
Mononuclear cell (%)	75.00 (32.00–90.00)	89.00 (75.00–95.00)	0.000	72.50 (40.00–90.00)	90.00 (71.50–96.00)	0.014
sCEA (ng/mL)	3.08 (1.66–9.75)	1.31 (0.90–2.37)	0.000	2.59 (1.28–5.90)	1.72 (1.01–2.82)	0.071
sCYFRA21-1 (ng/mL)	3.84 (2.19–9.09)	1.88 (1.29–2.81)	0.000	3.91 (2.27–7.61)	1.82 (1.32–2.16)	0.002
pCEA (ng/mL)	3.48 (0.98–93.19)	0.90 (0.56–1.59)	0.000	2.83 (0.87–68.60)	0.89 (0.60–1.78)	0.015
pCYFRA21-1 (ng/mL)	41.70 (14.80–188.00)	16.90 (8.11–39.90)	0.000	46.72 (13.80–178.00)	24.50 (18.10–58.25)	0.094

*p* < 0.05 is considered to have significant statistical difference. TB-IGRA, tuberculosis interferon-gamma release assays; pADA, pleural effusion adenosine deaminase; pLDH, lactate dehydrogenase; CEA, carcinoembryonic antigen; CYFRA21-1, cytokeratin 19 fragment.

### 3.2 Variable selection for the TPE prediction model

**FIGURE 1:**
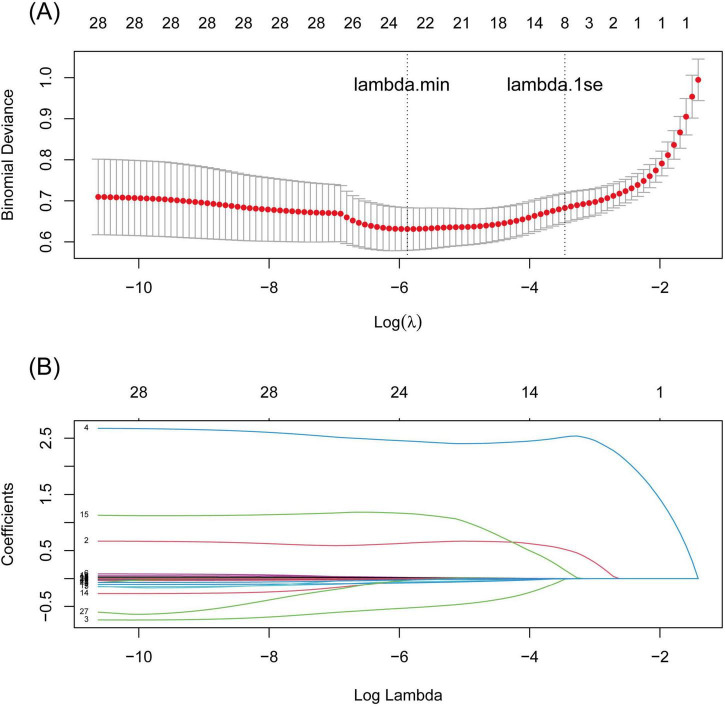
LASSO regression curves with 10-fold cross validation. **(A)** binomial deviance curve versus log(λ) based on the minimum criteria (left dotted line, λ = 0.004) and 1 SE criteria (right dotted line, λ = 0.035), **(B)** the coefficient profile plot against log(λ).

A total of 28 variables were significantly important in the univariate logistic analysis (Supplementary material 5) and were subsequently included in the LASSO binary logistic regression. Seven variables with non-zero coefficients were selected when the log(λ) mean squared error reached 1 standard error (SE) after 10-fold cross-validation (Figures 1A,B): fever, TB-IGRA, pADA, the mononuclear cell ratio, pLDH/pADA, pCEA, and pCYFRA21-1 concentration. According to the cutoff value based on the MSRS, four continuous variables, the mononuclear cell ratio and pLDH/pADA, pCEA, and pCYFRA21-1 concentrations, were converted into binary variables at cutoff values of 80%, 13.39, 3.58, and 89.2 ng/mL, respectively.

### 3.3 Construction of the TPE nomogram prediction model and scoring system

**FIGURE 2:**
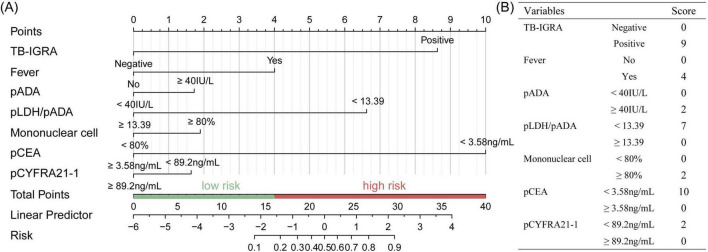
Establishment of the TPE scoring system in the training set. **(A)** Nomogram for TPE prediction of a patient with risk factors and the cutoff point was 16, **(B)** simplified scoring system and corresponding points of each variable.

**FIGURE 3:**
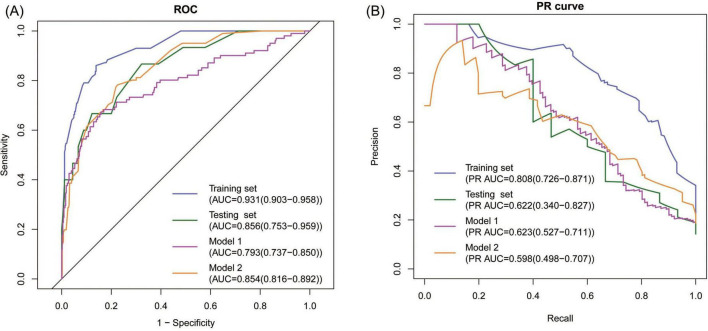
Discrimination of the model. **(A)** ROC analyses showing the AUC and 95% CI, **(B)** PR analyses showing the PR AUC and 95% CI.

The seven variables above were included to construct a nomogram model to discriminate high-risk individuals (Figure 2A). According to the ROC analysis of the nomogram model, the optimal cutoff point and risk were 15.687 points and 15.96%, as determined by the maximum Youden index, with a sensitivity of 0.860 (0.787–0.934), specificity of 0.861 (0.825–0.898), and AUC of 0.931 (0.903–0.958) (Figure 3A). For convenient risk assessment, the R package “nomogramFormula” outputted integer scores for each variable on the nomogram, with subsequent analyses using 16 points as the cutoff threshold (Figure 2B).

We also assessed the predictive performance of pADA, TB-IGRA, and their combination for TPE, and the AUCs were 0.811, 0.731, and 0.830, respectively, suggesting the superiority of the established model.

### 3.4 Internal evaluation of the new model

**FIGURE 4:**
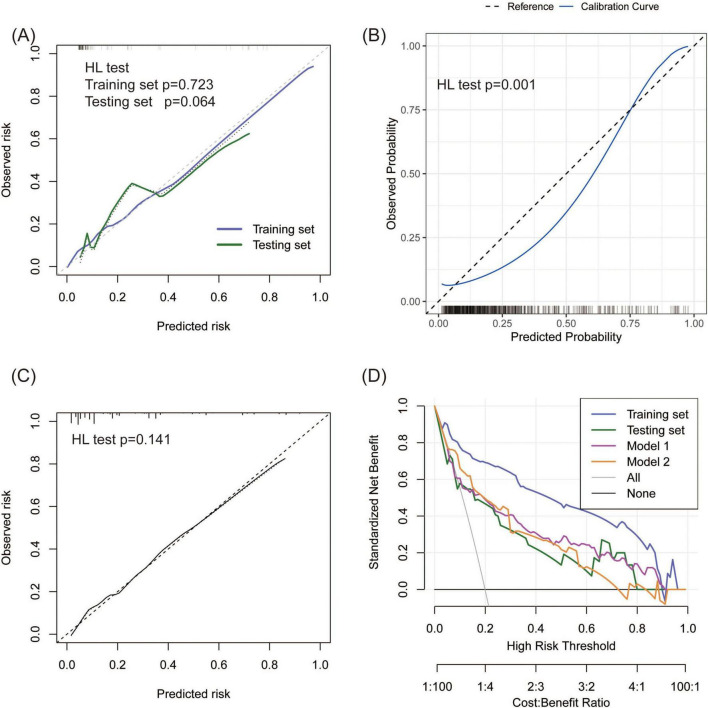
Consistency and practicability evaluation of TPE models. **(A–C)** Calibration curves for the new model, Model 1, and Model 2. A *p*-value < 0.05 in the HL test indicates a significant bias between the predicted and observed risk. **(D)** DCA comparison between the new model and two existing models on the basis of standardized net benefit. The high-risk thresholds for positive net benefits were 0.04–0.90 in the training set, 0.06–0.80 in the testing set, 0.09–0.90 in Model 1, and 0.06–0.80 in Model 2.

**FIGURE 5:**
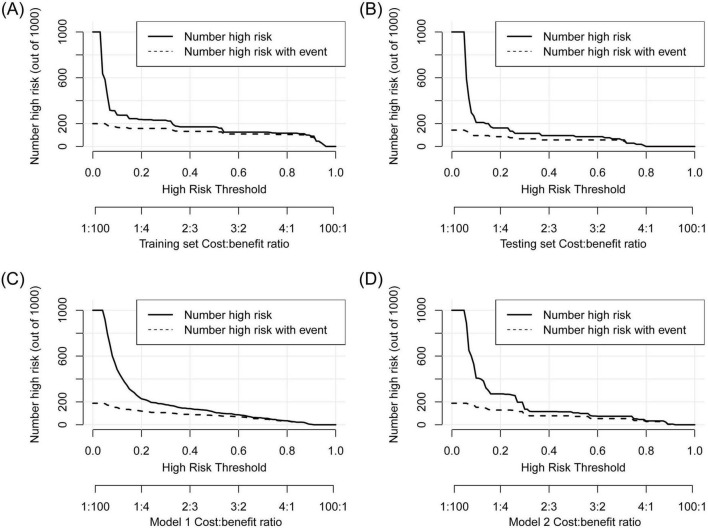
CIC analysis to show the gap between the predicted and observed TPE numbers at different high-risk thresholds. **(A)** Training set, **(B)** testing set, **(C)** Model 1, **(D)** Model 2. The TPE number determined by the models was highly matched to the actual TPE number when the risk threshold exceeded 0.2 in the three models.

First, ROC and PR analyses were conducted to clarify the discriminative ability of the model. In the testing set, the AUC was 0.856 (0.753–0.959), and the sensitivity and specificity were 0.667 (0.428–0.905) and 0.867 (0.796–0.937), respectively, when a cutoff point of ≥ 16 was used (Table 2). Owing to the prevalence rate imbalance in the dataset, the PR AUC values were 0.808 (0.726–0.871) and 0.622 (0.340–0.827) in the training and testing sets, respectively (Figure 3B). Second, the HL test confirmed the good consistency of the model, with *p*-values of 0.723 and 0.064 in the training and testing sets, respectively. As shown by the bootstrap (*n* = 1000) calibration curve, the predicted risk was relatively close to the observed risk (Figure 4A). The model achieved a brier score of 0.076 in the training set and 0.088 in the testing set, with calibration curve slopes of 1.000 and 0.647, intercepts of 0.000 and −0.590, and log-likelihood ratios of 217.643 and 21.024, respectively. Third, when the high-risk threshold was between 0.04 and 0.90 in the training set or between 0.06 and 0.80 in the testing set, this model demonstrated positive net benefits with a low curve slope (Figure 4D). The CIC showed that the TPE number determined by the nomogram was highly matched to the actual TPE number when the risk threshold exceeded 0.2 (Figures 5A,B).

**TABLE 2:** Diagnostic performance of new model, model 1, and model 2.

Metrics	New model			Model 1	Model 2
	Training set	Testing set	Validation set		
Cutoff	16 points			60% risk	11.038 points
ROC-AUC	0.931 (0.903–0.958)	0.856 (0.753–0.959)	0.925 (0.867–0.984)	0.793 (0.737–0.850)	0.854 (0.816–0.892)
Sensitivity	0.860 (0.787–0.934)	0.667 (0.428–0.905)	0.893 (0.778–1.000)	0.396 (0.301–0.491)	0.416 (0.320–0.512)
Specificity	0.861 (0.825–0.898)	0.867 (0.796–0.937)	0.868 (0.803–0.932)	0.970 (0.954–0.986)	0.954 (0.934–0.974)
Accuracy	0.861 (0.861–0.862)	0.838 (0.836–0.841)	0.873 (0.872–0.875)	0.862 (0.862–0.863)	0.853 (0.852–0.853)
NPV	0.961 (0.940–0.983)	0.940 (0.889–0.991)	0.968 (0.933–1.004)	0.874 (0.844–0.904)	0.876 (0.846–0.905)
PPV	0.607 (0.520–0.693)	0.455 (0.246–0.663)	0.641 (0.490–0.792)	0.755 (0.639–0.871)	0.677 (0.561–0.794)
NLR	0.162 (0.096–0.274)	0.385 (0.187–0.790)	0.123 (0.042–0.361)	0.623 (0.531–0.730)	0.612 (0.519–0.723)
PLR	6.203 (4.707–8.174)	5.000 (2.645–9.452)	6.760 (4.081–11.197)	13.283 (7.384–23.892)	9.065 (5.573–14.746)
NRI	–	–	–	−1.036 (−1.227 to −0.844), *p* < 0.001	−0.906 (−1.102 to −0.710), *p* < 0.001
IDI	–	–	–	−0.216 (−0.265 to −0.168), *p* < 0.001	−0.217 (−0.269 to −0.164), *p* < 0.001

ROC, receiver operating characteristic curves; AUC, area under the curve; NPV, negative predictive value; PPV, positive predictive value; NLR, negative likelihood ratio; PLR, positive likelihood ratio; NRI, net reclassification improvement; IDI, integrated discrimination improvement.

### 3.5 Comparison between the new model and existing models

TPE prediction models have been reported in several studies, but few studies have compared different models. This study compared the new model with two other TPE models on the basis of discrimination, consistency, and practicability. Model 1 included four parameters, namely, age, TB-IGRA, lnRMMPE, and pADA, with a cutoff risk of 60% (17). Model 2 included six variables, namely, age, sex, absence of cancer, TB-IGRA, pADA, and CRP, with a cutoff point of 11.038 (16). As shown in Figures 3A,B and Table 2, in this research population, Model 1 had an AUC of 0.793 (0.737–0.850), a PR AUC of 0.623 (0.527–0.711), a sensitivity of 0.396 (0.301–0.491), and a specificity of 0.970 (0.954–0.986). Model 2 had an AUC of 0.854 (0.816–0.892), a PR AUC of 0.598 (0.498–0.707), a sensitivity of 0.416 (0.320–0.512), and a specificity of 0.954 (0.934–0.974). In Model 1, the HL test yielded *p* = 0.001, and the calibration plot revealed bias between the predicted probability and the observed probability (Figure 4B). In Model 2, the *p*-value of the HL test was 0.141, and the calibration plot showed good consistency (Figure 4C). The brier scores were 0.115 and 0.109, the calibration slopes were 1.024 and 1.000, the intercepts were −0.487 and 0.000, and the log-likelihood ratios were 519.200 and 143.603 in Model 1 and Model 2, respectively. The high-risk threshold for positive net benefits was 0.09–0.90 in Model 1 and 0.06–0.80 in Model 2 (Figure 4D). The CIC did not differ from that of the new model (Figures 5C,D).

The net reclassification improvement (NRI) measures the improvement in classification accuracy of the target model, whereas the integrated discrimination improvement (IDI) quantifies the overall enhancement in predictive probability discrimination, together validating the discrimination improvement of the target model. The NRI and IDI were calculated to confirm the accuracy improvement in predicting TPE outcomes. Compared with our new model, Model 1 resulted in a continuous NRI of −1.036 (−1.227 to −0.844, *p* < 0.001) and an IDI of −0.216 (−0.265 to −0.168, *p* < 0.001), whereas Model 2 resulted in a continuous NRI of −0.906 (−1.102 to −0.710, *p* < 0.001) and an IDI of −0.217 (−0.269 to −0.164, *p* < 0.001) (Table 2).

### 3.6 External validation of the prediction model

**FIGURE 6:**
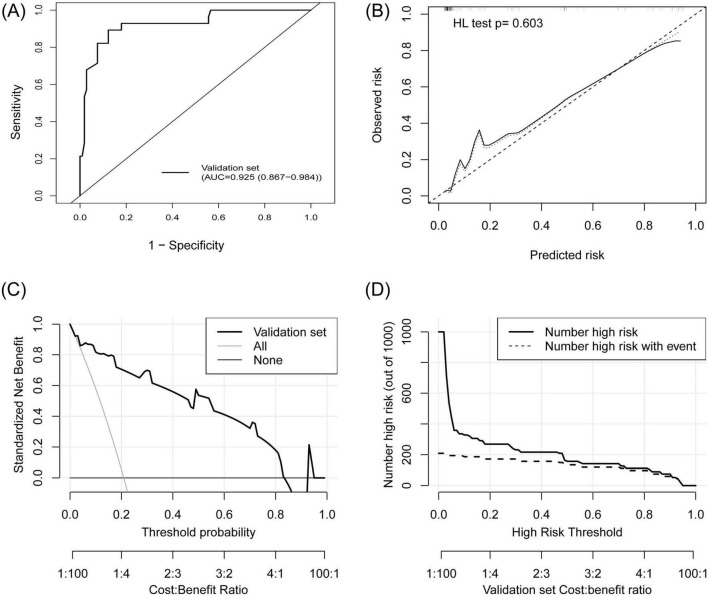
External validation of the TPE prediction model. **(A)** ROC curve, **(B)** calibration plot, **(C)** DCA showed that the high-risk threshold for positive net benefits was 0.03 to –0.83 in the external validation set, **(D)** CIC showed that the TPE number determined by the new model closely matched the actual TPE number in the external validation set.

A total of 134 patients (28 TPE patients and 106 non-TPE patients) were included in the external validation set. Among the non-TPE patients, 44 had MPE, and 62 had exudate pleural effusion caused by PPE or other reasons. The clinical characteristics of the patients in the external validation set are presented in Supplementary material 6. A comparison of the clinical characteristics between the training and external validation sets is shown in Supplementary material 7. TB-IGRA, sCEA, sCYFRA211, and pCYFRA21-1 showed statistically significant differences between the two datasets. In the external validation set, as shown in Figure 6A, the AUC was 0.925 (0.867–0.984), and the sensitivity and specificity were 0.893 (0.778–1.000) and 0.868 (0.803–0.932), respectively (Table 2). The *p*-value of the HL test was 0.603. The model demonstrated a brier score of 0.079, calibration slope of 1.036, intercept of −0.193, and log-likelihood ratio of 65.700. Figure 6B showed that the calibration curve of the prediction model roughly overlaps with the ideal calibration curve in the validation set. The model demonstrated positive net benefits when the high-risk threshold was between 0.03 and 0.83 in the validation set (Figure 6C). The CIC showed that the TPE number determined by the new model was closely matched to the actual TPE number in the validation set, especially when the risk threshold exceeded 0.2 (Figure 6D).

### 3.7 Decision threshold analysis and online risk calculator

**FIGURE 7:**
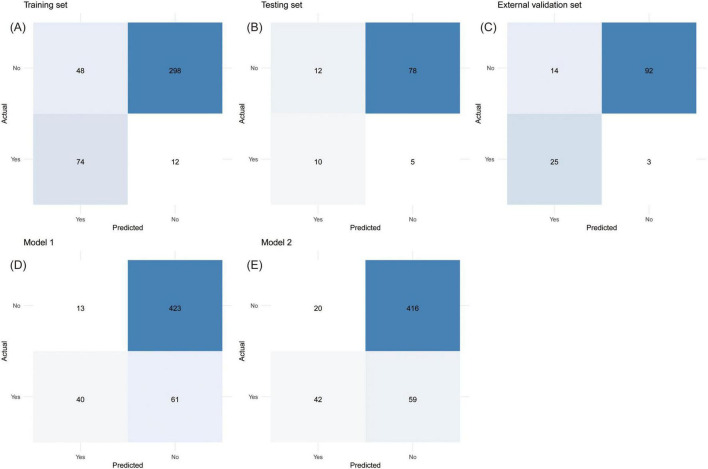
The confusion matrices with directly calculable sensitivity, specificity, false positive rate, false negative rate, positive predictive value, and negative predictive value. **(A)** Training set, **(B)** testing set, **(C)** external validation set, **(D)** Model 1, **(E)** Model 2.

The model performance in the training set at different thresholds was calculated, revealing that increased thresholds lead to decreased sensitivity and improved specificity, as shown in Table 3. Figures 7A–E showed the confusion matrices of the new model, Model 1, and Model 2 at the optimal threshold. To facilitate the risk calculation for individuals, a user-friendly online risk calculator was developed, which is available at https://tperiskprediction.shinyapps.io/TPE_risk_model/.

**TABLE 3:** Decision threshold analysis at different cutoff points in the training set.

Cutoff	14 points	15 points	16 points	17 points	18 points	19 points
Sensitivity	0.884 (0.816–0.951)	0.872 (0.802–0.943)	0.860 (0.787–0.934)	0.826 (0.745–0.906)	0.791 (0.705–0.877)	0.791 (0.705–0.877)
Specificity	0.815 (0.774–0.856)	0.827 (0.787–0.866)	0.861 (0.825–0.898)	0.864 (0.828–0.900)	0.893 (0.861–0.926)	0.905 (0.874–0.936)
Accuracy	0.829 (0.828–0.829)	0.836 (0.835–0.836)	0.861 (0.861–0.862)	0.856 (0.856–0.857)	0.873 (0.872–0.873)	0.882 (0.881–0.882)
NPV	0.966 (0.945–0.987)	0.963 (0.941–0.984)	0.961 (0.940–0.983)	0.952 (0.929–0.976)	0.945 (0.920–0.970)	0.946 (0.921–0.970)
PPV	0.543 (0.460–0.625)	0.556 (0.472–0.639)	0.607 (0.520–0.693)	0.602 (0.513–0.690)	0.648 (0.556–0.739)	0.673 (0.582–0.765)
NLR	0.143 (0.079–0.256)	0.155 (0.089–0.269)	0.162 (0.096–0.274)	0.202 (0.127–0.320)	0.234 (0.155–0.354)	0.231 (0.153–0.349)
PLR	4.778 (3.780–6.038)	5.029 (3.941–6.418)	6.203 (4.707–8.174)	6.078 (4.580–8.065)	7.394 (5.351–10.217)	8.290 (5.888–11.674)

NPV, negative predictive value; PPV, positive predictive value; NLR, negative likelihood ratio; PLR, positive likelihood ratio.

## 4 Discussion

TPE is a disease with a high incidence and disability rate that is difficult to diagnose. In this study, we constructed a simple and accessible new TPE model with good discrimination ability, consistency, and clinical effects on the basis of clinical characteristics, as well as biochemical indicators of serum and pleural effusion. The prediction performance of the new model was not inferior to that of the existing models.

The levels of the tumor markers CEA and CYFRA21-1 in pleural effusion fluid are often used to predict malignant pleural effusion (MPE) (22–25). Owing to the similar pleural effusion and serum biochemical indicators, as well as clinical symptoms, distinguishing between TPE and MPE is challenging. The use of tumor markers in pleural effusion fluid is a non-invasive way to differentiate TPE from MPE with improved accuracy. Ren et al. (26) used four machine learning algorithms to construct a TPE prediction model and found that pCEA is a significant predictor. Liu et al. (27) discovered that pCEA and pCYFRA21-1 are important features in the diagnosis of TPE.

ADA is a purine degradation enzyme present mostly in the lymphatic system and is linked to immune function and intracellular infections (28, 29). LDH is a cytoplasmic enzyme found in virtually all major organ systems. An elevated level of LDH in pleural effusion fluid typically indicates lung or pleural tissue damage and endothelial injury (30). The pLDH/pADA ratio is lower and the pADA is greater in TPE patients than in non-TPE patients, which has long been applied to distinguish TPE patients effectively from non-TPE patients (20, 29, 31). These studies employed pLDH/pADA cutoff thresholds within the range of 15–20, and the AUCs were between 0.94 and 0.97. Additionally, a systematic review recommended a pLDH/pADA cutoff value of 15 (32). Our study adopted a pLDH/pADA cutoff value of 13.39, which was basically consistent with prior studies.

In this study, the AUCs of the single variables pADA and TB-IGRA and their combination for predicting TPE were lower than those of the model based on seven variables. This suggested that neither the individual indicator nor their combination alone was optimal, necessitating the establishment of a multi-indicator integrated TPE model. Lei et al. (17) established a model with an AUC of 0.845–0.903, a sensitivity of 0.700–0.770, and a specificity of 0.880–0.920. Wu et al. (16) built a scoring model with an AUC of 0.992, a sensitivity of 0.937–0.929, and a specificity of 0.968–0.933. When these two prediction models were applied to our samples, the IDI and NRI confirmed that the new model obtained greater positive prediction accuracy. Compared with these two prediction models, the new model had better sensitivity, false negative rate, NPV, and NLR, but worse specificity, false positive rate and PLR. However, the disparity may be due to population characteristics, prevalence, and statistical methods, given that there was no significant difference in age, sex, or CRP in our data, which were the main predictors of Model 1 or Model 2.

Regarding the clinical implications of the model, the improved sensitivity and false negative rate of the new model suggest a lower missed diagnosis rate in tuberculosis-endemic or resource-limited regions, enhancing the detection rate of TPE. In a primary care setting where thoracoscopy is unavailable, this model could help identify candidates for empirical tuberculosis treatment. Additionally, a high NPV and lower NLR suggest that TPE is useful for ruling out TPE, making it suitable for initial screening in high-prevalence regions and areas with limited diagnostic access, where it can effectively identify low-risk patients who do not require further invasive examinations, thereby avoiding unnecessary procedures. Therefore, this model could be particularly helpful in community hospitals where invasive biopsy is not readily available. However, the increased false positive rate and reduced specificity leading to misdiagnosis may heighten healthcare burdens. Different endemic areas and healthcare resource settings can select appropriate risk thresholds on the basis of their specific circumstances to balance sensitivity and specificity for optimal screening efficacy.

Prediction models based on indicators from other studies also showed good diagnostic performance. Liu et al. (15, 27) utilized a large pleural effusion cohort to develop TPE models via logistic regression and a support vector machine, with AUCs of 0.937 and 0.914, sensitivities of 0.890 and 0.947, and specificities of 0.895 and 0.807, respectively. Ren et al. (26) developed a random forest model with an AUC of 0.965, a sensitivity of 0.906, and a specificity of 0.923 but lacked DCA and the CIC. Shu et al. (33) analyzed cytotoxic T lymphocyte-related cytokines and established a TPE diagnostic model on a small sample dataset, with an AUC of 0.920, a sensitivity of 0.829, and a specificity of 0.867. Although machine learning models exhibit higher AUCs, the nomogram model offers greater transparency with interpretable variables, facilitating clinical implementation. Since our data did not include the variables corresponding to these models, direct comparison with these models was difficult.

This study also has several limitations. First, the data were obtained retrospectively and from a single medical center, which may not be representative of the pleural effusion population in all regions. Additionally, including only Chinese populations may limit the model’s generalizability across regions with different demographic and genetic characteristics. Large multicenter prospective studies involving healthcare institutions of varying levels and ethnic groups will be essential to enhance the generalizability of our model. Second, the degradation in calibration and goodness-of-fit across both the testing set and the external validation set may be attributed to small sample sizes, insufficient positive events, and prevalence deviance. While LASSO regression was used to mitigate overfitting risks, a larger-scale dataset is essential for further validation of model performance. Third, owing to the inconsistency in patient examination items, the study did not collect enough laboratory indicators to validate additional models. In terms of statistical analysis, binarization of continuous variables may result in the loss of variable information and reduce the prediction accuracy of the model. The subgroup analyses are absent. Future research should conduct subgroup and stratification analyses by age, sex, MPE vs. PPE, etc., to further investigate the model’s predictive performance across different populations.

## 5 Conclusion

In summary, this study established a novel TPE model based on seven variables, fever, TB-IGRA, pADA, the mononuclear cell ratio, pLDH/pADA, pCEA, and pCYFRA21-1 concentration, with good diagnostic value and clinical efficacy. This approach may yield good clinical benefits and increase the detection rate of TPE.

## Data Availability

The data analyzed in this study is subject to the following licenses/restrictions: The datasets used and/or analyzed during the current study are available from the corresponding author on reasonable request. Requests to access these datasets should be directed to Yongchun Shen, shen_yongchun@126.com.
