# Supplementary material for: Development and validation of a prediction model based on a nomogram for tuberculous pleural effusion
Source: Front Med (Lausanne). 2025 Jul 18;12:1589406. doi: 10.3389/fmed.2025.1589406 (PMC12313491; doi:10.3389/fmed.2025.1589406)
Supplement: Supplementary file 2 [file Data_Sheet_2.docx]

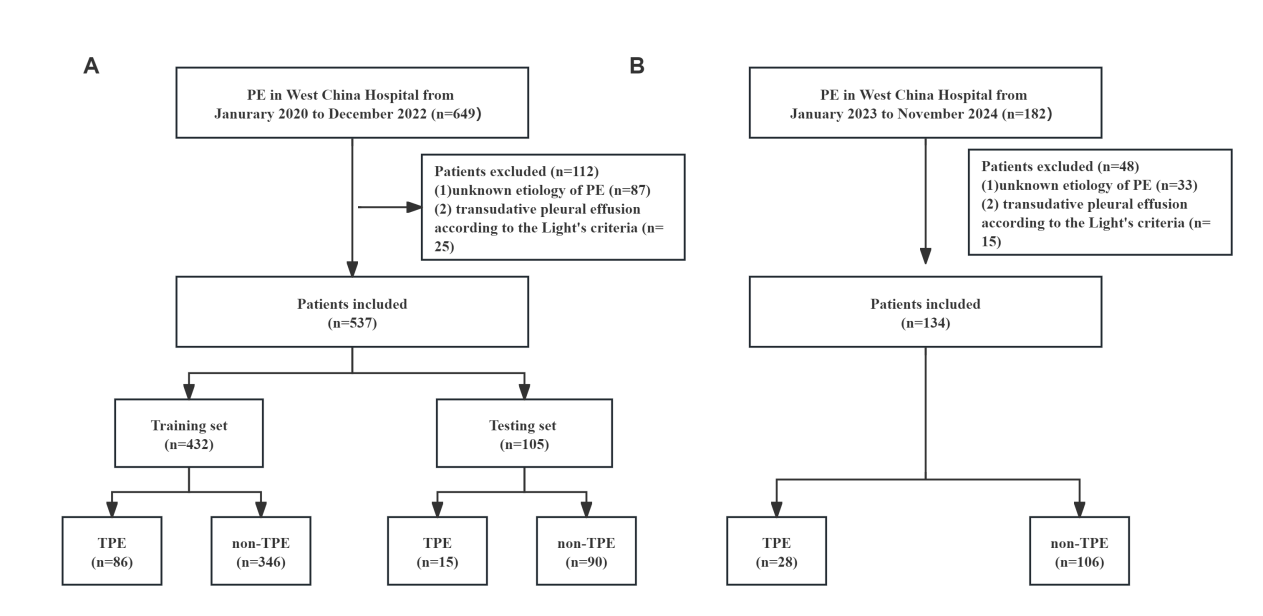


Supplemental Material 2. The fowchart of patient selection.(A) Derivation population. (B) External validation population.
