# Supplementary material for: Development and validation of a prediction model based on a nomogram for tuberculous pleural effusion
Source: Front Med (Lausanne). 2025 Jul 18;12:1589406. doi: 10.3389/fmed.2025.1589406 (PMC12313491; doi:10.3389/fmed.2025.1589406)
Supplement: Supplementary file 3 [file Data_Sheet_3.docx]

Supplemental Material 3

Comparison of characteristics between training and testing set.

| Variables | Training set (N=432) | Testing set (N=105) | Total (N=537) | p |
| --- | --- | --- | --- | --- |
| TPE |  |  |  | 0.186 |
| No | 346 (80.09%) | 90 (85.71%) | 436 (81.19%) |  |
| Yes | 86 (19.91%) | 15 (14.29%) | 101 (18.81%) |  |
| Sex |  |  |  | 0.431 |
| Female | 163 (37.73%) | 44 (41.90%) | 207 (38.55%) |  |
| Male | 269 (62.27%) | 61 (58.10%) | 330 (61.45%) |  |
| Smoking |  |  |  | 0.599 |
| No | 272 (62.96%) | 69 (65.71%) | 341 (63.50%) |  |
| Yes | 160 (37.04%) | 36 (34.29%) | 196 (36.50%) |  |
| Fever |  |  |  | 0.727 |
| No | 356 (82.41%) | 85 (80.95%) | 441 (82.12%) |  |
| Yes | 76 (17.59%) | 20 (19.05%) | 96 (17.88%) |  |
| Hemoptysis |  |  |  | 0.952 |
| No | 415 (96.06%) | 101 (96.19%) | 516 (96.09%) |  |
| Yes | 17 (3.94%) | 4 (3.81%) | 21 (3.91%) |  |
| Dyspnea |  |  |  | 0.260 |
| No | 312 (72.22%) | 70 (66.67%) | 382 (71.14%) |  |
| Yes | 120 (27.78%) | 35 (33.33%) | 155 (28.86%) |  |
| Cough with sputum |  |  |  | 0.401 |
| No | 129 (29.86%) | 27 (25.71%) | 156 (29.05%) |  |
| Yes | 303 (70.14%) | 78 (74.29%) | 381 (70.95%) |  |
| Chest pain |  |  |  | 0.614 |
| No | 303 (70.14%) | 71 (67.62%) | 374 (69.65%) |  |
| Yes | 129 (29.86%) | 34 (32.38%) | 163 (30.35%) |  |
| TB-IGRA |  |  |  | 0.118 |
| Negative | 341 (78.94%) | 90 (85.71%) | 431 (80.26%) |  |
| Positive | 91 (21.06%) | 15 (14.29%) | 106 (19.74%) |  |
| pADA ≥ 40 (U/L) |  |  |  | 0.104 |
| No | 374 (86.57%) | 97 (92.38%) | 471 (87.71%) |  |
| Yes | 58 (13.43%) | 8 (7.62%) | 66 (12.29%) |  |
| Age (year) | 64.00 (52.00-73.00) | 61.00 (52.00-69.00) | 64.00 (52.00-72.00) | 0.207 |
| HGB (g/L) | 122.00 (104.00-134.50) | 123.00 (105.00-137.00) | 122.00 (104.00-135.00) | 0.370 |
| PLT (×10^9^/L) | 241.00 (171.00-311.50) | 252.00 (173.00-321.00) | 243.00 (172.00-314.00) | 0.443 |
| WBC (×10^9^/L) | 7.04 (5.30-9.66) | 7.04 (5.56-9.33) | 7.04 (5.37-9.56) | 0.544 |
| Neutrophil (×10^9^/L) | 4.58 (3.16-6.79) | 4.85 (3.30-6.79) | 4.67 (3.16-6.79) | 0.689 |
| Lymphocyte (×10^9^/L) | 0.98 (0.66-1.37) | 1.09 (0.78-1.44) | 1.02 (0.69-1.39) | 0.107 |
| NLR | 4.05 (2.71-7.82) | 4.11 (2.30-7.34) | 4.05 (2.65-7.65) | 0.507 |
| sTP (g/L) | 63.85 (58.30-69.80) | 64.10 (59.50-69.60) | 63.90 (58.60-69.80) | 0.891 |
| sALB (g/L) | 34.90 (30.70-39.50) | 35.30 (31.50-40.10) | 35.00 (30.80-39.60) | 0.442 |
| sGLB (g/L) | 28.50 (24.35-33.10) | 27.80 (23.90-32.50) | 28.30 (24.20-33.10) | 0.487 |
| sLDH (IU/L) | 188.50 (154.50-243.50) | 181.00 (155.00-227.00) | 186.00 (155.00-241.00) | 0.283 |
| Mononuclear cell (%) | 80.00 (38.50-92.00) | 75.00 (45.00-90.00) | 80.00 (40.00-92.00) | 0.973 |
| Multinuclear cell (%) | 14.00 (5.00-41.00) | 12.00 (5.00-43.00) | 14.00 (5.00-42.00) | 0.823 |
| lnRMMPE | 1.74 (-0.02-2.94) | 1.73 (0.12-2.92) | 1.73 (0.06-2.94) | 0.854 |
| pTP (g/L) | 40.45 (32.65-47.30) | 42.40 (32.90-48.30) | 40.70 (32.80-47.60) | 0.351 |
| pLDH (IU/L) | 299.00 (170.50-616.00) | 271.00 (195.00-511.00) | 292.00 (181.00-603.00) | 0.843 |
| pALB (g/L) | 23.30 ± 7.22 | 24.12 ± 7.53 | 23.46 ± 7.28 | 0.301 |
| pADA (IU/L) | 10.15 (7.00-20.10) | 11.30 (7.70-16.40) | 10.20 (7.10-19.20) | 0.843 |
| pLDH/pADA | 31.59 (18.23-51.38) | 27.04 (17.58-55.42) | 30.83 (18.18-52.50) | 0.670 |
| sCEA (ng/mL) | 2.63 (1.31-6.81) | 2.20 (1.23-5.25) | 2.54 (1.28-6.27) | 0.378 |
| sCA199 (U/mL) | 11.40 (6.04-28.65) | 10.60 (5.94-21.60) | 11.30 (6.02-25.30) | 0.596 |
| sCA125 (U/mL) | 99.00 (46.95-202.10) | 81.30 (43.40-199.20) | 94.80 (45.90-199.20) | 0.516 |
| sCYFRA21-1 (ng/mL) | 3.34 (1.90-7.19) | 3.37 (1.82-6.18) | 3.36 (1.88-7.17) | 0.915 |
| sNSE (ng/mL) | 13.10 (10.35-19.00) | 13.20 (11.00-17.90) | 13.20 (10.60-18.70) | 0.643 |
| pCEA (ng/mL) | 1.96 (0.79-31.22) | 2.08 (0.80-31.60) | 1.96 (0.79-31.60) | 0.950 |
| pCA199 (U/mL) | 4.72 (2.00-14.50) | 4.12 (2.00-16.90) | 4.59 (2.00-15.60) | 0.863 |
| pCA125 (U/mL) | 698.00 (271.00-1243.50) | 627.00 (224.00-1219.00) | 676.00 (263.00-1239.00) | 0.271 |
| pCYFRA21-1 (ng/mL) | 32.36 (12.70-151.00) | 44.30 (14.90-149.00) | 34.40 (13.00-151.00) | 0.371 |
| pNSE (ng/mL) | 8.09 (3.87-26.00) | 7.85 (4.63-15.20) | 8.02 (4.04-23.60) | 0.898 |
| PCT (ng/mL) | 9.29 (0.38-73.30) | 8.44 (0.13-69.20) | 8.90 (0.31-72.90) | 0.616 |
| CRP (mg/L) | 24.70 (12.00-71.76) | 23.10 (8.91-77.60) | 24.40 (11.00-73.60) | 0.710 |
| IL-6 (ng/mL) | 0.13 (0.04-3.52) | 0.14 (0.03-4.58) | 0.13 (0.04-3.66) | 0.350 |
| FDP (mg/L) | 6.60 (3.10-14.75) | 8.50 (4.10-14.10) | 6.90 (3.20-14.40) | 0.227 |
| D-dimer (mg/L FEU) | 2.54 (1.26-6.27) | 3.73 (1.37-7.51) | 2.70 (1.31-6.71) | 0.179 |
| p/sCEA | 0.86 (0.53-3.02) | 0.97 (0.61-4.03) | 0.89 (0.55-3.07) | 0.340 |
| p/sCA199 | 0.51 (0.28-1.00) | 0.53 (0.33-1.00) | 0.51 (0.28-1.00) | 0.426 |
| p/sCA125 | 5.89 (2.24-12.52) | 4.97 (2.09-12.07) | 5.87 (2.21-12.44) | 0.447 |
| p/sCYFRA21-1 | 10.16 (3.65-25.32) | 9.38 (3.78-27.88) | 10.08 (3.68-25.42) | 0.848 |
| p/sNSE | 0.54 (0.27-1.71) | 0.55 (0.31-1.01) | 0.55 (0.28-1.51) | 0.863 |
| p/sTP | 0.63 (0.54-0.71) | 0.63 (0.57-0.71) | 0.63 (0.55-0.71) | 0.395 |
| p/sLDH | 1.31 (0.86-2.72) | 1.42 (1.01-2.19) | 1.36 (0.87-2.67) | 0.678 |
| p/sALB | 0.68 (0.58-0.76) | 0.70 (0.60-0.76) | 0.68 (0.58-0.76) | 0.312 |
