# Supplementary material for: Development and validation of a prediction model based on a nomogram for tuberculous pleural effusion
Source: Front Med (Lausanne). 2025 Jul 18;12:1589406. doi: 10.3389/fmed.2025.1589406 (PMC12313491; doi:10.3389/fmed.2025.1589406)
Supplement: Supplementary file 4 [file Data_Sheet_4.docx]

Supplemental Material 4

Details of baseline characteristics of variables in training set and testing set.

| Variables | Training set |  |  | Testing set |  |  |
| --- | --- | --- | --- | --- | --- | --- |
|  | non-TPE(n=346) | TPE (n=86) | p | non-TPE (n=90) | TPE (n=15) | p |
| Sex |  |  | 0.176 |  |  | 0.872 |
| Female | 136 (39.31%) | 27 (31.40%) |  | 38 (42.22%) | 6 (40.00%) |  |
| Male | 210 (60.69%) | 59 (68.60%) |  | 52 (57.78%) | 9 (60.00%) |  |
| Smoking |  |  | 0.337 |  |  | 0.502 |
| No | 214 (61.85%) | 58 (67.44%) |  | 58 (64.44%) | 11 (73.33%) |  |
| Yes | 132 (38.15%) | 28 (32.56%) |  | 32 (35.56%) | 4 (26.67%) |  |
| Fever |  |  | 0.000 |  |  | 0.128 |
| No | 302 (87.28%) | 54 (62.79%) |  | 75 (83.33%) | 10 (66.67%) |  |
| Yes | 44 (12.72%) | 32 (37.21%) |  | 15 (16.67%) | 5 (33.33%) |  |
| Hemoptysis |  |  | 0.036 |  |  | 0.405 |
| No | 329 (95.09%) | 86 (100.00%) |  | 86 (95.56%) | 15 (100.00%) |  |
| Yes | 17 (4.91%) | 0 ( 0.0%) |  | 4 (4.44%) | 0 ( 0.0%) |  |
| Dyspnea |  |  | 0.295 |  |  | 1.000 |
| No | 246 (71.10%) | 66 (76.74%) |  | 60 (66.67%) | 10 (66.67%) |  |
| Yes | 100 (28.90%) | 20 (23.26%) |  | 30 (33.33%) | 5 (33.33%) |  |
| Cough with sputum |  |  | 0.028 |  |  | 0.236 |
| No | 95 (27.46%) | 34 (39.53%) |  | 25 (27.78%) | 2 (13.33%) |  |
| Yes | 251 (72.54%) | 52 (60.47%) |  | 65 (72.22%) | 13 (86.67%) |  |
| Ches tpain |  |  | 0.079 |  |  | 0.496 |
| No | 236 (68.21%) | 67 (77.91%) |  | 62 (68.89%) | 9 (60.00%) |  |
| Yes | 110 (31.79%) | 19 (22.09%) |  | 28 (31.11%) | 6 (40.00%) |  |
| TB-IGRA |  |  | 0.000 |  |  | 0.000 |
| Negative | 316 (91.33%) | 25 (29.07%) |  | 82 (91.11%) | 8 (53.33%) |  |
| Positive | 30 (8.67%) | 61 (70.93%) |  | 8 (8.89%) | 7 (46.67%) |  |
| pADA ≥ 40 (IU/L) |  |  | 0.000 |  |  | 0.000 |
| No | 311 (89.88%) | 63 (73.26%) |  | 87 (96.67%) | 10 (66.67%) |  |
| Yes | 35 (10.12%) | 23 (26.74%) |  | 3 (3.33%) | 5 (33.33%) |  |
| Age (year) | 64.00 (53.00-72.00) | 57.50 (46.00-74.00) | 0.153 | 64.00 (54.00-69.00) | 56.00 (46.50-65.00) | 0.140 |
| HGB (g/L) | 122.00 (102.00-134.00) | 121.50 (108.00-136.00) | 0.650 | 121.59 ± 25.88 | 121.20 ± 22.56 | 0.956 |
| PLT (×10^9^/L) | 239.00 (171.00-311.00) | 244.50 (177.00-314.00) | 0.693 | 248.50 (172.00-321.00) | 293.00 (180.00-315.50) | 0.567 |
| WBC (×10^9^/L) | 7.24 (5.62-10.32) | 6.15 (4.84-7.50) | 0.000 | 7.54 (6.09-10.07) | 5.92 (4.68-6.59) | 0.001 |
| Neutrophil (×10^9^/L) | 4.77 (3.34-7.25) | 3.94 (2.82-5.24) | 0.002 | 4.96 (3.52-7.70) | 3.14 (2.50-4.86) | 0.020 |
| Lymphocyte (×10^9^/L) | 1.00 (0.68-1.35) | 0.94 (0.62-1.46) | 0.858 | 1.10 (0.78-1.50) | 1.05 (0.77-1.25) | 0.339 |
| NLR | 4.11 (2.75-8.62) | 3.71 (2.68-6.51) | 0.089 | 4.42 (2.37-7.52) | 3.01 (2.20-6.03) | 0.250 |
| sTP (g/L) | 63.73 ± 8.02 | 65.86 ± 8.08 | 0.028 | 64.21 ± 9.83 | 63.17 ± 7.46 | 0.696 |
| sALB (g/L) | 34.80 (30.70-39.50) | 34.95 (30.70-39.60) | 0.730 | 35.33 ± 5.77 | 35.21 ± 6.12 | 0.938 |
| sGLB (g/L) | 28.05 (24.10-32.70) | 30.55 (25.40-35.70) | 0.016 | 27.75 (23.90-33.20) | 27.80 (25.65-29.30) | 0.805 |
| sLDH (IU/L) | 193.00 (158.00-260.00) | 176.50 (146.00-211.00) | 0.005 | 179.50 (149.00-227.00) | 186.00 (171.00-235.00) | 0.320 |
| Mononuclear cell (%) | 75.00 (32.00-90.00) | 89.00 (75.00-95.00) | 0.000 | 72.50 (40.00-90.00) | 90.00 (71.50-96.00) | 0.014 |
| Multinuclear cell (%) | 15.00 (5.00-53.00) | 10.00 (4.00-24.00) | 0.008 | 15.00 (5.00-46.00) | 8.00 (2.50-17.00) | 0.039 |
| lnRMMPE | 1.66 (-0.41-2.89) | 2.20 (1.10-2.94) | 0.004 | 1.40 (-0.20-2.87) | 2.44 (1.56-3.52) | 0.021 |
| pTP (g/L) | 39.50 (31.90-46.70) | 43.35 (36.40-50.40) | 0.014 | 42.40 (31.30-48.70) | 44.80 (37.65-45.60) | 0.714 |
| pLDH (IU/L) | 320.00 (182.00-639.00) | 229.00 (146.00-400.00) | 0.014 | 287.00 (200.00-557.00) | 234.00 (169.50-314.00) | 0.171 |
| pALB (g/L) | 23.23 ± 7.27 | 23.59 ± 7.05 | 0.681 | 24.13 ± 7.86 | 24.08 ± 5.29 | 0.982 |
| pADA (IU/L) | 8.95 (6.60-14.10) | 24.75 (10.10-42.90) | 0.000 | 10.10 (7.70-15.20) | 25.70 (9.10-46.70) | 0.030 |
| pLDH/pADA | 36.33 (22.82-58.33) | 12.41 (7.53-21.73) | 0.000 | 32.03 (20.00-64.42) | 11.00 (8.55-22.37) | 0.000 |
| sCEA (ng/mL) | 3.08 (1.66-9.75) | 1.31 (0.90-2.37) | 0.000 | 2.59 (1.28-5.90) | 1.72 (1.01-2.82) | 0.071 |
| sCA199 (U/mL) | 12.80 (6.57-40.70) | 7.38 (4.09-13.10) | 0.000 | 12.51 (6.13-25.00) | 6.97 (2.94-9.45) | 0.005 |
| sCA125 (U/mL) | 103.55 (48.50-211.00) | 72.87 (42.90-163.00) | 0.035 | 89.15 (44.70-203.00) | 60.20 (40.70-170.00) | 0.420 |
| sCYFRA21-1 (ng/mL) | 3.84 (2.19-9.09) | 1.88 (1.29-2.81) | 0.000 | 3.91 (2.27-7.61) | 1.82 (1.32-2.16) | 0.002 |
| sNSE (ng/mL) | 13.20 (10.50-20.20) | 12.80 (10.20-16.80) | 0.233 | 14.10 (11.10-19.00) | 11.80 (9.34-14.45) | 0.037 |
| pCEA (ng/mL) | 3.48 (0.98-93.19) | 0.90 (0.56-1.59) | 0.000 | 2.83 (0.87-68.60) | 0.89 (0.60-1.78) | 0.015 |
| pCA199 (U/mL) | 5.68 (2.10-30.84) | 2.71 (2.00-4.92) | 0.000 | 5.81 (2.00-64.40) | 2.70 (2.00-5.03) | 0.065 |
| pCA125 (U/mL) | 730.00 (332.00-1319.00) | 521.50 (149.30-1090.00) | 0.005 | 636.00 (244.00-1312.00) | 271.00 (116.60-855.00) | 0.157 |
| pCYFRA21-1 (ng/mL) | 41.70 (14.80-188.00) | 16.90 (8.11-39.90) | 0.000 | 46.72 (13.80-178.00) | 24.50 (18.10-58.25) | 0.094 |
| pNSE (ng/mL) | 8.96 (3.87-27.70) | 6.73 (3.70-13.80) | 0.075 | 8.72 (4.85-16.96) | 5.05 (3.12-8.14) | 0.025 |
| PCT (ng/mL) | 9.29 (0.41-71.50) | 9.37 (0.14-77.30) | 0.773 | 9.41 (0.13-74.50) | 4.40 (0.78-11.68) | 0.218 |
| CRP (mg/L) | 25.73 (12.50-77.60) | 20.46 (10.20-54.20) | 0.123 | 25.70 (8.91-79.20) | 17.40 (7.47-67.60) | 0.504 |
| IL-6 (ng/mL) | 0.13 (0.04-3.17) | 0.12 (0.04-9.56) | 0.702 | 0.20 (0.03-4.58) | 0.05 (0.02-2.60) | 0.214 |
| FDP (mg/L) | 5.80 (2.80-15.30) | 8.35 (5.40-13.50) | 0.058 | 7.40 (3.60-14.40) | 10.20 (6.05-13.75) | 0.511 |
| D-dimer (mg/L FEU) | 2.31 (1.20-6.06) | 3.57 (2.10-6.53) | 0.047 | 3.44 (1.35-7.51) | 5.27 (2.94-7.04) | 0.266 |
| p/sCEA | 1.12 (0.56-5.54) | 0.65 (0.50-0.81) | 0.000 | 1.02 (0.61-5.07) | 0.67 (0.56-1.00) | 0.047 |
| p/sCA199 | 0.51 (0.27-1.32) | 0.50 (0.32-0.80) | 0.380 | 0.55 (0.32-1.09) | 0.52 (0.36-0.95) | 0.916 |
| p.sCA125 | 6.14 (2.31-12.76) | 4.28 (1.88-10.87) | 0.096 | 5.42 (2.09-12.07) | 3.96 (1.83-14.63) | 0.759 |
| p/sCYFRA21-1 | 10.18 (3.65-25.73) | 9.13 (3.37-22.67) | 0.517 | 9.18 (3.02-28.09) | 13.46 (6.05-26.03) | 0.472 |
| p/sNSE | 0.55 (0.26-1.76) | 0.46 (0.28-1.03) | 0.340 | 0.57 (0.32-1.03) | 0.42 (0.30-0.63) | 0.215 |
| p/sTP | 0.62 (0.53-0.70) | 0.67 (0.56-0.74) | 0.040 | 0.63 (0.57-0.72) | 0.67 (0.59-0.70) | 0.773 |
| p/sLDH | 1.33 (0.84-2.77) | 1.29 (0.88-2.19) | 0.778 | 1.45 (1.02-2.49) | 1.05 (0.74-1.79) | 0.174 |
| p/sALB | 0.68 (0.58-0.76) | 0.69 (0.60-0.76) | 0.419 | 0.69 (0.59-0.78) | 0.71 (0.62-0.74) | 0.773 |
