# Supplementary material for: Development and validation of a prediction model based on a nomogram for tuberculous pleural effusion
Source: Front Med (Lausanne). 2025 Jul 18;12:1589406. doi: 10.3389/fmed.2025.1589406 (PMC12313491; doi:10.3389/fmed.2025.1589406)
Supplement: Supplementary file 5 [file Data_Sheet_5.docx]

Supplemental Material 5

Univariate logistic analysis of variables in training set.

| Variables | β | S.E | Z | *P* | OR (95%CI) |
| --- | --- | --- | --- | --- | --- |
| Age | -0.02 | 0.01 | -2.25 | 0.025 | 0.98 (0.97 - 0.99) |
| HGB | 0.00 | 0.00 | 0.78 | 0.435 | 1.00 (0.99 - 1.01) |
| PLT | 0.00 | 0.00 | 0.37 | 0.713 | 1.00 (1.00 - 1.00) |
| WBC | -0.15 | 0.04 | -3.60 | <.001 | 0.86 (0.79 - 0.93) |
| Neutrophil | -0.12 | 0.04 | -2.97 | 0.003 | 0.89 (0.82 - 0.96) |
| Lymphocyte | -0.02 | 0.14 | -0.12 | 0.902 | 0.98 (0.74 - 1.30) |
| NLR | -0.06 | 0.02 | -2.30 | 0.022 | 0.94 (0.90 - 0.99) |
| sTP | 0.03 | 0.02 | 2.18 | 0.029 | 1.03 (1.01 - 1.07) |
| sALB | 0.01 | 0.02 | 0.44 | 0.658 | 1.01 (0.97 - 1.05) |
| sGLB | 0.04 | 0.02 | 2.20 | 0.028 | 1.04 (1.01 - 1.07) |
| sALB/sGLB | -0.48 | 0.34 | -1.39 | 0.165 | 0.62 (0.32 - 1.22) |
| sLDH | -0.01 | 0.00 | -2.69 | 0.007 | 0.99 (0.99 - 0.99) |
| Mononuclear cell | 0.02 | 0.00 | 3.98 | <.001 | 1.02 (1.01 - 1.03) |
| Multinuclear cell | -0.01 | 0.00 | -2.54 | 0.011 | 0.99 (0.98 - 0.99) |
| lnRMMPE | 0.18 | 0.06 | 2.87 | 0.004 | 1.20 (1.06 - 1.36) |
| pTP | 0.02 | 0.01 | 1.91 | 0.056 | 1.02 (1.00 - 1.04) |
| pLDH | -0.00 | 0.00 | -1.50 | 0.133 | 1.00 (1.00 - 1.00) |
| pALB | 0.01 | 0.02 | 0.41 | 0.680 | 1.01 (0.97 - 1.04) |
| pLDH/pADA | -0.07 | 0.01 | -6.67 | <.001 | 0.93 (0.91 - 0.95) |
| sCEA | -0.35 | 0.08 | -4.44 | <.001 | 0.70 (0.60 - 0.82) |
| sCA199 | -0.01 | 0.00 | -1.98 | 0.047 | 0.99 (0.99 - 0.99) |
| sCA125 | -0.01 | 0.00 | -2.05 | 0.041 | 0.99 (0.99 - 0.99) |
| sCYFRA21-1 | -0.03 | 0.02 | -1.70 | 0.090 | 0.97 (0.95 - 1.00) |
| sNSE | -0.04 | 0.02 | -2.16 | 0.031 | 0.97 (0.94 - 0.99) |
| pCEA | -0.39 | 0.11 | -3.51 | <.001 | 0.68 (0.55 - 0.84) |
| pCA199 | -0.01 | 0.00 | -2.45 | 0.014 | 0.99 (0.99 - 0.99) |
| pCA125 | -0.01 | 0.00 | -2.81 | 0.005 | 0.99 (0.99 - 0.99) |
| pCYFRA21-1 | -0.01 | 0.00 | -3.92 | <.001 | 0.99 (0.99 - 0.99) |
| pNSE | -0.01 | 0.00 | -2.41 | 0.016 | 0.99 (0.98 - 0.99) |
| PCT | -0.00 | 0.00 | -0.45 | 0.651 | 1.00 (1.00 - 1.00) |
| CRP | -0.01 | 0.00 | -2.34 | 0.019 | 0.99 (0.99 - 0.99) |
| IL-6 | -0.01 | 0.00 | -1.42 | 0.157 | 0.99 (0.99 - 1.00) |
| FDP | -0.00 | 0.01 | -0.29 | 0.771 | 1.00 (0.98 - 1.02) |
| D-dimer | -0.00 | 0.02 | -0.15 | 0.878 | 1.00 (0.96 - 1.04) |
| p/sCEA | -1.03 | 0.27 | -3.84 | <.001 | 0.36 (0.21 - 0.60) |
| p/sCA199 | -0.27 | 0.12 | -2.25 | 0.024 | 0.76 (0.61 - 0.97) |
| p/sCA125 | -0.01 | 0.01 | -1.29 | 0.197 | 0.99 (0.97 - 1.01) |
| p/sCYFRA21-1 | 0.00 | 0.00 | 0.51 | 0.612 | 1.00 (1.00 - 1.01) |
| p/sNSE | -0.12 | 0.06 | -1.87 | 0.061 | 0.89 (0.78 - 1.01) |
| p/sTP | 0.91 | 0.79 | 1.17 | 0.244 | 2.50 (0.54 - 11.64) |
| p/sLDH | -0.01 | 0.01 | -1.16 | 0.248 | 0.99 (0.97 - 1.01) |
| p/sALB | 0.20 | 0.74 | 0.27 | 0.791 | 1.22 (0.28 - 5.20) |
| Sex |  |  |  |  |  |
| Female |  |  |  |  | 1.00 (Reference) |
| Male | 0.35 | 0.26 | 1.35 | 0.177 | 1.42 (0.85 - 2.34) |
| Smoking |  |  |  |  |  |
| No |  |  |  |  | 1.00 (Reference) |
| Yes | -0.25 | 0.26 | -0.96 | 0.337 | 0.78 (0.47 - 1.29) |
| Fever |  |  |  |  |  |
| No |  |  |  |  | 1.00 (Reference) |
| Yes | 1.40 | 0.28 | 5.10 | <.001 | 4.07 (2.37 - 6.98) |
| Hemoptysis |  |  |  |  |  |
| No |  |  |  |  | 1.00 (Reference) |
| Yes | -15.22 | 581.98 | -0.03 | 0.979 | 0.00 (0.00 - Inf) |
| Dyspnea |  |  |  |  |  |
| No |  |  |  |  | 1.00 (Reference) |
| Yes | -0.29 | 0.28 | -1.04 | 0.297 | 0.75 (0.43 - 1.29) |
| Cough with sputum |  |  |  |  |  |
| No |  |  |  |  | 1.00 (Reference) |
| Yes | -0.55 | 0.25 | -2.18 | 0.030 | 0.58 (0.35 - 0.95) |
| Chest pain |  |  |  |  |  |
| No |  |  |  |  | 1.00 (Reference) |
| Yes | -0.50 | 0.28 | -1.75 | 0.081 | 0.61 (0.35 - 1.06) |
| TB -IGRA |  |  |  |  |  |
| Negative |  |  |  |  | 1.00 (Reference) |
| Positive | 3.25 | 0.30 | 10.65 | <.001 | 25.70 (14.14 - 46.71) |
| pADA ≥ 40 |  |  |  |  |  |
| No |  |  |  |  | 1.00 (Reference) |
| Yes | 1.18 | 0.30 | 3.90 | <.001 | 3.24 (1.80 - 5.86) |
