# Supplementary material for: Development and validation of a prediction model based on a nomogram for tuberculous pleural effusion
Source: Front Med (Lausanne). 2025 Jul 18;12:1589406. doi: 10.3389/fmed.2025.1589406 (PMC12313491; doi:10.3389/fmed.2025.1589406)
Supplement: Supplementary file 6 [file Data_Sheet_6.docx]

Supplemental Material 6

The clinical characteristics of the external validation set.

| TPE | non-TPE (N=106) | TPE (N=28) | Total (N=134) | p |
| --- | --- | --- | --- | --- |
| Sex |  |  |  | 0.297 |
| Female | 36 (33.96%) | 6 (21.43%) | 42 (31.34%) |  |
| Male | 70 (66.04%) | 22 (78.57%) | 92 (68.66%) |  |
| TB-IGRA |  |  |  | 0.000 |
| Negative | 92 (86.79%) | 2 (7.14%) | 94 (70.15%) |  |
| Positive | 14 (13.21%) | 26 (92.86%) | 40 (29.85%) |  |
| Fever |  |  |  | 0.000 |
| No | 89 (83.96%) | 14 (50.00%) | 103 (76.87%) |  |
| Yes | 17 (16.04%) | 14 (50.00%) | 31 (23.13%) |  |
| pADA ≥ 40 (IU/L) |  |  |  | 0.081 |
| No | 92 (86.79%) | 20 (71.43%) | 112 (83.58%) |  |
| Yes | 14 (13.21%) | 8 (28.57%) | 22 (16.42%) |  |
| Age (year) | 61.00 (51.00-71.00) | 62.00 (55.50-72.00) | 61.00 (51.00-71.00) | 0.700 |
| pADA (IU/L) | 8.25 (5.10-19.00) | 25.40 (10.30-44.85) | 10.00 (5.70-25.60) | 0.001 |
| pLDH (IU/L) | 285.00 (146.00-927.00) | 233.00 (130.00-461.00) | 274.00 (146.00-804.00) | 0.671 |
| pLDH/pADA | 41.61 (26.00-75.40) | 9.78 (6.90-20.48) | 32.50 (17.84-66.70) | 0.000 |
| Mononuclear cell (%) | 68.00 (12.00-90.00) | 94.50 (83.50-96.50) | 78.50 (16.00-90.00) | 0.000 |
| sCEA (ng/mL) | 2.23 (1.10-5.00) | 1.59 (0.85-2.60) | 2.01 (1.02-4.22) | 0.030 |
| sCYFRA211 (ng/mL) | 3.17 (1.69-4.79) | 2.19 (1.18-3.24) | 2.60 (1.64-4.73) | 0.094 |
| pCEA (ng/mL) | 2.46 (0.82-306.00) | 1.30 (0.26-3.53) | 2.09 (0.65-21.90) | 0.017 |
| pCYFRA21-1 (ng/mL) | 22.00 (7.43-98.40) | 28.50 (9.08-98.40) | 24.25 (8.22-98.40) | 0.390 |

p<0.05 is considered to have significant statistical difference.

TB-IGRA, tuberculosis interferon-gamma release assays; pADA, pleural effusion adenosine deaminase; pLDH, lactate dehydrogenase; CEA, carcinoembryonic antigen; CYFRA21-1, cytokeratin 19 fragment.
