# Supplementary material for: Development and validation of a prediction model based on a nomogram for tuberculous pleural effusion
Source: Front Med (Lausanne). 2025 Jul 18;12:1589406. doi: 10.3389/fmed.2025.1589406 (PMC12313491; doi:10.3389/fmed.2025.1589406)
Supplement: Supplementary file 7 [file Data_Sheet_7.docx]

Supplemental Material 7

The comparison of clinical characteristics between the training and external validation set.

| Dataset | Training set (N=432) | External validation set (N=134) | p |
| --- | --- | --- | --- |
| TPE |  |  | 0.900 |
| No | 346 (80.09%) | 106 (79.10%) |  |
| Yes | 86 (19.91%) | 28 (20.90%) |  |
| Sex |  |  | 0.214 |
| Female | 163 (37.73%) | 42 (31.34%) |  |
| Male | 269 (62.27%) | 92 (68.66%) |  |
| TB-IGRA |  |  | 0.047 |
| Negative | 341 (78.94%) | 94 (70.15%) |  |
| Positive | 91 (21.06%) | 40 (29.85%) |  |
| Fever |  |  | 0.192 |
| No | 356 (82.41%) | 103 (76.87%) |  |
| Yes | 76 (17.59%) | 31 (23.13%) |  |
| pADA ≥ 40 (IU/L) |  |  | 0.467 |
| No | 374 (86.57%) | 112 (83.58%) |  |
| Yes | 58 (13.43%) | 22 (16.42%) |  |
| Age (year) | 64.00 (52.00-73.00) | 61.00 (51.00-71.00) | 0.447 |
| pADA (IU/L) | 10.15 (7.00-20.10) | 10.00 (5.70-25.60) | 0.421 |
| pLDH (IU/L) | 299.00 (170.50-616.00) | 274.00 (146.00-804.00) | 0.230 |
| pLDH/pADA | 31.59 (18.23-51.38) | 32.50 (17.84-66.70) | 0.272 |
| Mononuclear cell (%) | 80.00 (38.50-92.00) | 78.50 (16.00-90.00) | 0.201 |
| sCEA (ng/mL) | 2.63 (1.31-6.81) | 2.01 (1.02-4.22) | 0.011 |
| sCYFRA211 (ng/mL) | 3.34 (1.90-7.19) | 2.60 (1.64-4.73) | 0.003 |
| pCEA (ng/mL) | 1.96 (0.79-31.22) | 2.09 (0.65-21.90) | 0.615 |
| pCYFRA21-1 (ng/mL) | 32.36 (12.70-151.00) | 24.25 (8.22-98.40) | 0.029 |

p<0.05 is considered to have significant statistical difference.

TB-IGRA, tuberculosis interferon-gamma release assays; pADA, pleural effusion adenosine deaminase; pLDH, lactate dehydrogenase; CEA, carcinoembryonic antigen; CYFRA21-1, cytokeratin 19 fragment.
